# Safety of Vitamin D Food Fortification and Supplementation: Evidence from Randomized Controlled Trials and Observational Studies

**DOI:** 10.3390/foods10123065

**Published:** 2021-12-09

**Authors:** Folasade A. Adebayo, Suvi T. Itkonen, Taina Öhman, Mairead Kiely, Kevin D. Cashman, Christel Lamberg-Allardt, on behalf of the ODIN Consortium

**Affiliations:** 1 Calcium Research Unit, Department of Food and Nutrition, University of Helsinki, P.O. Box 66, FI-00014 Helsinki, Finland; folasade.adebayo@helsinki.fi (F.A.A.); suvi.itkonen@helsinki.fi (S.T.I.); taina.ohman@gmail.com (T.Ö.); 2 Cork Centre for Vitamin D and Nutrition Research, School of Food and Nutritional Sciences, University College Cork, T12 Y337 Cork, Ireland; M.Kiely@ucc.ie (M.K.); K.Cashman@ucc.ie (K.D.C.)

**Keywords:** vitamin D, (bio)fortification, supplementation, safety, adverse health effect, serum 25(OH)D, serum calcium, *ODIN*

## Abstract

The safety considerations of food-based solutions for vitamin D deficiency prevention, such as fortification and supplementation, are critical. On the basis of collective data from 20 randomized controlled trials (RCTs) and 20 national healthy surveys, as well as prospective cohort studies (PCSs) across the *ODIN* project (“Food-based solutions for optimal vitamin D nutrition and health through the life cycle”, FP7-613977), we analyzed the potential safety issues arising from vitamin D intakes and/or supplementation. These adverse consequences included high serum 25-hydroxyvitamin D (S-25(OH)D) concentrations (>125 nmol/L), high serum calcium concentrations, and vitamin D intakes in excess of the tolerable upper intake levels (ULs). In the RCTs (*n* = 3353, with vitamin D doses from 5–175 µg/day), there were no reported adverse effects. The prevalence of high S-25(OH)D was <10% when vitamin D supplements were administered, and <0.1% for fortified foods. Elevated serum calcium was observed among <0.5% in both administration types. No *ODIN* RCT participants exceeded the age-specific ULs. In observational studies (*n* = 61,082), the prevalence of high 25(OH)D among children/adolescents, adults, and older adults was <0.3%, with no evidence of adverse effects. In conclusion, high S-25(OH)D concentrations >125 nmol/L were rare in the RCTs and PCSs, and no associated adverse effects were observed.

## 1. Introduction

Vitamin D is important for musculoskeletal health, and its extraskeletal roles are played in the cellular, endocrine, immune, cardiovascular, and other systems [1,2]. Hence, vitamin D deficiency/insufficiency manifests as rickets and osteomalacia and has also been associated with some extraskeletal diseases, such as cardiovascular disease and type 2 diabetes, among others [1,2]. Vitamin D is derived from diets and through cutaneous synthesis in human skin upon exposure to ultraviolet B (UVB) from sunlight [3]. However, the numbers of naturally occurring vitamin-D-rich foods, containing either vitamin D_3_ (cholecalciferol) or D_2_ (ergocalciferol), are limited [3,4]. Thus, vitamin D food fortification has been the most suitable approach for enhancing vitamin D intake and status in the general population [4]. This is important, especially for the people living in northern latitudes, where there is limited or no cutaneous vitamin D production for several months during wintertime because of the inadequate levels of UVB irradiation for the synthesis [5]. Besides vitamin D food fortification, which is the traditional way of adding vitamin D directly to food product, the vitamin D content of food can be increased through biofortification, without the direct exogenous addition [4]. Examples of such biofortification include the increase in the vitamin D_2_ content of mushrooms by UVB-exposure that induces vitamin D production [6], and the increase in the vitamin D_3_ content of eggs through vitamin D-rich hen feed [7], among others [8]. In addition, studies have clearly demonstrated the effectiveness of oral supplementation in improving vitamin D status (i.e., increasing serum 25-hydroxyvitamin D (S-25(OH)D) concentrations), especially among individuals with low statuses [9,10,11]. Either from foods or supplements, vitamin D_2_ is generally considered less potent than vitamin D_3_ in raising and maintaining S-25(OH)D concentrations [12,13].

Safety is always an important consideration, not only when formulating recommendations for nutrient intakes, but also in establishing strategies aimed at preventing deficiencies in the population, such as food fortification and dietary supplementation [14]. The Institute of Medicine (IOM) and the European Food Safety Authority (EFSA) have both evaluated the potential for high intakes of vitamin D to produce adverse effects and have, accordingly, set tolerable upper intake levels (ULs) for vitamin D [15,16,17]. These ULs were established on the basis of minimizing the risk of hypercalcemia (high serum calcium), using evidence from vitamin D supplementation studies. High serum 25(OH)D concentrations (>220 nmol/L) may lead to hypercalcemia [18]. While hypercalcemia is mainly related to primary hyperparathyroidism or malignancy, it can also be induced by very high calcium or vitamin D intakes. Hypercalcemia can be life-threatening, and its symptoms include: neuropsychiatric manifestations, such as apathy, confusion, depression, irritability, hallucinations, and, in extreme cases, stupor, and coma; gastrointestinal symptoms, such as nausea, vomiting, anorexia, and constipation; cardiovascular manifestations, such as ectopy and hypertension; and renal symptoms, such as polyuria, polydipsia, dehydration, and renal colic from the passage of renal stones [19,20]. Orally ingested vitamin D is relatively safe, and toxicity is not apparent at doses of up to 250 µg/day [15,17] in healthy adults. However, to maximize public health protection, both agencies applied an uncertainty factor of 1.2 to 2 in order to ensure no risk for harm [15,16]. A UL for vitamin D of 100 µg/day was assigned for all adults, including pregnant and lactating women, whereas there were slight variations in the two institutions for the ULs for children [15,16,17] (see Supplementary Table S1).

The International Agency for Research on Cancer (IARC) pointed out that there are no data on the health hazards of maintaining high serum 25(OH)D in healthy persons over long periods and urged caution to be mindful of past experiences with other compounds and treatments (e.g., some antioxidants and hormone replacement therapies) that showed serious adverse effects when chronic high-dose supplements were used [21]. Over and above the risk of hypercalcemia, reports of U-shaped and reverse J-shaped distributions have emerged for serum 25(OH)D and the adverse consequences, including all-cause mortality, cardiovascular disease risk, parathyroid hormone (PTH) suppression, and fetal growth restriction [15,22,23], which deserve serious consideration by both researchers and authoritative agencies. For these reasons, the IOM issued an additional cautionary note in the recommendations on the potential adverse effects of a sustained serum 25(OH)D above ~125–150 nmol/L, acknowledging the lack of empirical data [15]. Such serum concentrations could potentially be achieved at vitamin D intakes below the ULs.

In terms of monitoring the safety of any dietary strategies to increase vitamin D intakes in the population, the main requirement is that intakes at the 97.5th percentile of the distribution do not exceed the UL for a specified age group. In the European Union-funded *ODIN* (“Food-based solutions for optimal vitamin D nutrition and health through the life cycle”, FP7-613977) vitamin D project (2013–2017), different food-based strategies (such as the fortification and biofortification of different foods to accommodate the diverse dietary practices in modern society), aimed at increasing the habitual vitamin D intakes in the population, were proposed and evaluated [24]. These solutions sought to change the shape of the vitamin D intake distribution, increasing the median to ~10 µg/day, without increasing the intake to the UL [25]. In addition, the effects of higher serum 25(OH)D on various health outcomes were also investigated in observational and vitamin D supplement intervention studies within *ODIN* [24]. Importantly, the project maintained a watching safety brief over all activities with respect to exposure, status, and health outcomes in order to ensure the protection of public health and safety. The aim of this paper is to provide a summary of the key findings of the *ODIN* project as they relate to safety considerations in relation to increasing vitamin D intake and serum 25(OH)D concentrations. These safety considerations were based on collective data from 20 randomized controlled trials (RCTs) and 20 national healthy surveys, as well as prospective cohort studies (PCS) across the *ODIN* project. Details of the selection of the included studies are provided elsewhere [24].

## 2. Methods, including Safety Approach, within the *ODIN* Project

The overall aim of the *ODIN* vitamin D project was to develop effective, safe, and sustainable solutions to prevent vitamin D deficiency and improve vitamin D-related health outcomes using a food-first approach, as described in detail elsewhere [24]. Structurally, the project, based on a collaborative and multidisciplinary consortium of 30 partners from 19 countries, had nine research work packages (WPs; akin to mini-projects; see Supplementary Figure S1), all of which had a particular focus aligned with the project’s key aims. The main aim of WP9 (the Safety WP) was to document all of the safety issues (see below) across the project and its various WPs, which included data from vitamin D RCTs, PCSs, and national nutrition surveys. The approach for the selection of these RCTs and PCSs within the *ODIN* project has been outlined elsewhere [20], but, in brief, the studies represented those RCTs or PCSs that fit with the specific aims of the individual WPs within the project, but also, importantly, those that had biobanked serum samples to facilitate standardization or the reanalysis of 25(OH)D by LC-MS/MS in order to ensure the removal of any method-related differences in the data. This was critical, as the 25(OH)D data was pooled for individual participant data analyses of the vitamin D and nonskeletal health effects.

### 2.1. Specification of Safety and the Adverse Effects

#### 2.1.1. High Serum 25-Hydroxyvitamin D

Safety, in terms of high serum 25(OH)D, was defined as the prevalence of serum 25(OH)D >125 nmol/L. This aligns with the Institute of Medicine (IOM), 2011 [15], which, in setting their tolerable upper levels (ULs) for vitamin D intakes, also considered if the intakes of vitamin D were likely to lead to serum 25(OH)D concentrations in excess of approximately 125 to 150 nmol/L. They considered that this might be of concern on the basis of some of the observed U-shaped or reverse J-shaped relationships between serum 25(OH)D and mortality, as well as other health outcomes [4]. However, the European Food Safety Agency (EFSA) found no studies with an association between vitamin D intake and an increased risk for adverse long-term health outcomes, and that studies reporting on an association between 25(OH)D concentration and all-cause mortality or cancer were inconsistent [16].

All of the serum 25(OH)D concentration data within *ODIN* were either retrospectively standardized, as per the *Vitamin D Standardization Program* (VDSP) [26], fully reanalyzed, or analyzed de novo using the *ODIN* core analytical platform for serum total 25(OH)D at University College Cork [27], which was certified by the Centers for Disease Control and Prevention (CDC). The specificity of this certified LC-MS/MS platform avoids the artificially high measurement values for serum 25(OH)D generated by some immuno-assays, especially for concentrations >100 nmol/L [28]. This is important in the context of a more valid comparison of the prevalence data on high-serum 25(OH)D concentrations across populations and countries.

The IOM’s caution about serum 25(OH)D >125 nmol/L relates to sustained concentrations, as opposed to the more transient elevations that are part of the normal seasonal fluctuations in serum 25(OH)D. Blood sampling in several of the population samples (RCTs and observational studies) within *ODIN* was done throughout the year. Hence, yearly prevalence estimates of serum 25(OH)D >125 nmol/L would include data from the summer sampling, when higher serum 25(OH)D concentrations are more likely to be achieved [29]. During the subsequent winter and spring, serum 25(OH)D concentrations will significantly decline, as the UVB availability for the synthesis of previtamin D_3_ in the skin becomes absent or limited [5]. High concentrations of serum 25(OH)D achieved in wintertime are more likely due to high dietary intake (i.e., vitamin D supplement use and/or the use of vitamin-D-fortified foods in addition to natural food sources). Therefore, in addition to yearly prevalence estimates, the prevalence of serum 25(OH)D >125 nmol/L during an extended wintertime (October–March), which might be more indicative of sustained high concentrations, are also reported in the present work.

#### 2.1.2. High Serum Calcium Concentration

The normal range of serum calcium is 2.1–2.6 mmol/L, with concentrations ≥2.6 mmol/L typically defined as hypercalcemia [15,17]. The upper limit of the reference range for Ca (mmol/L), however, varies slightly, depending on the method of analysis and the laboratory. Hence, hypercalcemia is defined in the present work as a serum calcium concentration >2.50–2.75 mmol/L, on the basis of laboratory-specific values.

#### 2.1.3. Dietary Intakes of Vitamin D Exceeding the Tolerable Upper Level

Safety, in terms of dietary vitamin D intakes, was defined as the risk of exceeding the EFSA age-specific UL values, namely, 25 µg/day for infants ≤6 months, and 35 µg/day for infants aged 6–12 months [17], as well as 50 µg/day for children aged 1–10 years, and 100 µg/day for children and adolescents aged 11–17 years and adults [16]. Additionally, in the case of vitamin D RCTs within *ODIN*, we used a crude stratification, depending on the dose of the supplemental vitamin D_3_ being ≤70 µg/day or ≥71 µg/day, being representative of “low to moderately high” or “moderately high to high” doses, respectively.

### 2.2. Studies Contributing Data on Safety and Adverse Effects

Safety surveillance data emanated from numerous RCTs within *ODIN*’s WPs 4–6 and 8, as well as observational studies within WPs 1, 2, and 8 (Supplementary Figure S1), as follows.

#### 2.2.1. Randomized Controlled Trials

Safety data from a total of 20 RCTs arising from WPs 4–6 and 8 were included (Table 1). Of these, 7 were de novo RCTs performed within *ODIN* (total *n* = 736 subjects), all of which used supplemental vitamin D and/or vitamin-D-(bio)fortified foods. The project had one additional RCT using controlled UV-exposure (WP3), but the primary data from this study has not yet been published and, thus, is not included in the present work. Moreover, 12 previously conducted RCTs were used in ODIN’s individual participant data (IPD) meta-analysis focused on the beneficial or harmful effects of vitamin D_3_ on cardiovascular disease (CVD) and diabetes [30]. In this case, biobanked sera (baseline and endpoint) from the 12 studies (total combined *n* = 2617 subjects) were reanalyzed by the *ODIN* CDC-certified LC-MS/MS method [31,32,33,34,35,36,37,38,39,40,41,42,43,44,45,46,47,48,49,50,51,52,53,54].

In some of the RCTs, vitamin D_3_ supplements were administered once or twice a week, monthly, or quarterly, and the doses were reported in the form of international units (IUs) [38,42,43,46,47,48]. For this work, these were converted to daily dose equivalents and expressed in micrograms (i.e., 40 IU = 1 µg). A food frequency questionnaire was used in the studies that reported habitual dietary intakes of vitamin D; such data were not available from all RCTs. For those studies that did report habitual vitamin D intakes, these were added to the supplemental vitamin D to derive “total vitamin D intake” estimates.

#### 2.2.2. Observational Studies

VDSP standardized 25(OH)D data, and other safety data from 20 observational studies [55,56,57,58,59,60,61,62,63,64,65,66,67,68,69,70,71,72,73,74,75,76,77], were gathered from representative childhood/teenage and adult/older adult European populations (WP1) [78], as well as two pregnancy cohorts [79,80] and one infant [64,65] cohort (WP7) (Supplementary Table S2). In addition, 25(OH)D data from a second *ODIN* IPD-level meta-analysis, in this case, of the association between vitamin D and all-cause mortality (including cardiovascular and cancer mortality), based on eight previous PCSs (*n* = 26,916) [81], were included. Seven of these PCSs had their serum 25(OH)D data standardized by the VDSP protocols, whereas one had its serum 25(OH)D values measured de novo.

#### 2.2.3. Adverse Health Effects of High Vitamin D Intake or Serum 25(OH)D Concentrations

Safety, in terms of the additional potential adverse health effects of high vitamin D intake or serum 25(OH)D concentrations, was defined within the *ODIN* project as the risk of increased mortality, cardiovascular mortality, adverse pregnancy outcomes, any adverse fetal and infant outcomes, and an increased risk of eczema, asthma, and food allergies. Specifically, in the IPD meta-analysis of 12 RCTs to investigate whether there are beneficial or harmful effects of vitamin D_3_ supplementation [30], the specified outcomes were blood pressure, low-density lipoprotein (LDL), high-density lipoprotein (HDL), total cholesterol, triglycerides, PTH, glycated hemoglobin (HbA1c), fasting glucose, insulin and C-peptide, and 2-h glucose.

### 2.3. Collection of Safety Data

The data regarding the safety and the adverse effects throughout the *ODIN* project were collected via a specific questionnaire-based Excel repository platform. The questions on the safety considerations reflected the abovementioned specified measures, namely, the prevalence of high serum 25(OH)D concentrations, the prevalence of elevated serum calcium, new information on the possible associated adverse health effects, vitamin D intakes at, or in excess of, the ULs, when available, as well as other specific safety considerations.

### 2.4. Ethical Considerations

*ODIN* included a dedicated ethics work package, which collected the ethical approvals from pre-existing studies to ensure that the necessary consent was obtained to conduct 25(OH)D, and associated, data analysis. Ethical approval was obtained for the individual studies conducted within *ODIN* from the respective local ethical review boards [34,35,36,37,52,53,54].

## 3. Results

### 3.1. Study Characteristics

#### 3.1.1. Observational Setting

The characteristics of the observational studies included in this work are described in Supplementary Table S2. These studies were nationally representative nutrition surveys and epidemiological cohorts among a wide range of European populations across several countries. Surveys were conducted among infants and children, adolescents, adults, and older adults; cohorts among pregnant women and infant populations; and the IPD-level meta-analyses of cohorts among older adults.

#### 3.1.2. Interventional Setting

Table 1 shows the characteristics of the 20 vitamin D RCTs and their participants. The studies were all conducted in the following European countries: three each in Denmark, Ireland, and the Netherlands; five in Norway; two in Finland; and one each in the U.K., Greece, Germany, and Austria. One study each was conducted among the following population groups: children, adolescents, and pregnant women. Among the remaining 17 RCTs, all carried out in adult populations (age range 18–80 years), two studies were in women of ethnic groups, two in mixed gender populations with immigrant backgrounds, one among the elderly living in nursing home, and two among individuals with high BMI. One study each was carried out in a population with the following conditions: impaired glucose tolerance (IGT) and/or impaired fasting glucose (IFG), low bone mineral density, and arterial hypertension. The study durations varied from one to twelve months.

The vitamin D supplementation RCTs administered vitamin D_3_ supplements (18 RCTs) or vitamin D_2_ supplements (2 RCTs) (Table 1). The administered supplemental vitamin D_3_ doses ≤70 µg/day were: 5 µg/day; 10 µg/day; 15 µg/day; 20 µg/day; 25 µg/day; 30 µg/day; and 70 µg/day. Those of doses ≥71 µg/day (or the daily equivalent) were: 71 µg/day (i.e., 20,000 IU/week); 143 µg/day (i.e., 20,000 IU/twice a week or 40,000 IU/week); 163 µg/day (i.e., 800 IU/day + 20,000 IU/twice a week); and 175 µg/day. Each of the 2 RCTs that administered supplemental vitamin D_2_ used doses of 25 and 100 µg/d, respectively. Of the 4 RCTs administering vitamin D as fortified food(s), 1 RCT each used bread, cheese, mushrooms, or a combination of four different foods (vitamin D-fortified low-fat cheese, yoghurt, eggs, and crisp bread). Of the food-based RCTs, 2 used vitamin D_3_-fortified food(s) (i.e., cheese and combined fortified foods providing 5.7 and 20 µg/day, respectively), while the other 2 studies used vitamin D_2_-fortified foods (i.e., bread and mushrooms providing 25 and 100 µg/day, respectively) in the intervention arms.

### 3.2. Dietary Intakes of Vitamin D Exceeding the Tolerable Upper Level: Interventional Setting

In the supplemental vitamin D RCTs, the mean total vitamin D intakes were 25.3 and 29.7 µg/day at the 99th percentile for children and adolescents, respectively (data not shown). Among those RCTs conducted in adult populations, where habitual vitamin D intakes were reported (4 out of 6 de novo RCTs), the mean total intake was between 53.4 and 79.5 µg/day at the 99th percentile. In food-based vitamin D RCTs, the mean total intake was 70.7 µg/day at the 99th percentile (intervention with vitamin D_3_-enriched food) and 55.5 at the 97.5th percentile (intervention with vitamin D_2_-fortified bread). Vitamin D intakes did not exceed the UL (i.e., 50 µg/day for children aged 1–10 years; 100 µg/day for adolescents and adults) in any RCT with available data on habitual vitamin D intakes.

### 3.3. Prevalence of High Serum 25(OH)D Concentrations (>125 nmol/L): Observational and Interventional Setting

#### 3.3.1. Observational Studies in Children, Adolescents, and Older Adults

There were no cases of serum 25(OH)D concentrations >125 nmol/L during extended winter in seven of the eight European childhood and adolescent population samples [78]. In one child cohort (2-year-olds from the Cork BASELINE birth cohort) [64], three subjects (0.4%) had serum 25(OH)D concentrations >125 nmol/L during extended winter (Supplementary Table S2). Of the six European adult/older adult population samples, four had no cases of serum 25(OH)D concentrations >125 nmol/L during extended winter, and the prevalence in the other two studies (*Tromsø study—6th Survey* and the *New Hoorn Study*) [70,71,72,73] was 0.3%. In the one ethnic adult population sample, based in Finland (the *Maamu study*), the prevalence of serum 25(OH)D concentrations >125 nmol/L was 0.2% during extended winter.

Data from the eight prospective cohorts of older adults (median age 61.6 years, 58% females, combined *n* = 26,916) [81] showed that the prevalence of 25(OH)D >125 nmol/L ranged from 0 to 0.6% throughout the year, and from 0 to 0.3% during the extended wintertime (Supplementary Table S2).

Taking all 20 observational study samples of children/adolescents, adults, and older adults combined, out of a total of 61,082 subjects, 132 (0.22%) had serum 25(OH)D >125 nmol/L on a yearly basis, including the summer months, and only 70 during winter alone.

The prevalence of high 25(OH)D concentrations was almost zero in the pregnancy cohorts analyzed.

#### 3.3.2. Interventional Setting: Vitamin D Supplementation RCTs

The prevalence of serum 25(OH)D >125 nmol/L in the vitamin D RCT studies, stratified by supplemental doses ≤70 µg/day or ≥71 µg/day, is shown in Table 2. Of the 10 RCTs of children, adolescents, and adults with doses ≤70 µg/day (ranging from 5 to 70 µg/day), only 2 RCTs had cases of endpoint serum 25(OH)D >125 nmol/L, and only one subject in each (one adolescent and one adult woman of East African descent, both of whom received a vitamin D_3_ supplement of dose 20 µg/day for five months of extended winter in the U.K. and Finland, respectively (Table 2)). It is important to note that when habitual vitamin D intake was accounted for, the total vitamin D intakes of these two individuals were 23.2 and 59.5 µg/day, respectively (data not shown). In the vitamin D RCT among pregnant women, endpoint serum 25(OH)D >125 nmol/L was observed among eleven participants, of whom five women received 10 µg, and six women received a 20-µg vitamin D_3_ supplement daily for 25 weeks (none were in the placebo group, eight had endpoint sampling in summer (total vitamin D intakes in the range of 16.0–43.9 µg/day); three in winter (total vitamin D intakes in the range of 29.6–53.4 µg/day)).

All 6 of the RCTs that administered supplement doses of vitamin D_3_ ≥71 µg/day had cases of serum 25(OH)D >125 nmol/L, ranged from 7.0% in the groups receiving 71 µg/day of vitamin D_3_ to 91.9% in those receiving 163 µg/day (Table 2). These RCTs ranged in duration from six months to 1 year so were not winter-based trials only. Of note, however, in all but one of these RCTs, the placebo group had no cases of endpoint serum 25(OH)D >125 nmol/L. Two individuals in the placebo group of the trial of Norwegian adults with impaired glucose tolerance had serum 25(OH)D >125 nmol/L (no data available other than supplemental vitamin D intake).

#### 3.3.3. Interventional Setting: Vitamin D-Fortified Food-Based RCTs

In 3 out of the 4 RCTs using vitamin D-fortified foods, which provided doses of 5.7 µg vitamin D_3_/day, or 25 or 100 µg vitamin D_2_/day, no participants had endpoint serum 25(OH)D concentrations >125 nmol/L (Table 2). In the wintertime food-based RCT (in Denmark), 1 out of 35 women of Pakistani descent (2.9%), and 1 out of 37 Caucasian women of Danish descent (2.7%) who received vitamin D_3_-enriched food with 20 µg/day for 3 months had endpoint serum 25(OH)D >125 nmol/L. Accounting for habitual vitamin D intake together with supplemental vitamin D, their total vitamin D intakes were 25.8 and 27.4 µg/day, respectively.

In summary, out of 3353 participants who took part in the 20 vitamin D RCTs included in this *ODIN* safety work, a total of 320 (9.5%) individuals (belonging to the intervention arms that received either supplements or fortified foods) had serum 25(OH)D >125 nmol/L at the endpoint of the study. The proportion of the endpoint serum 25(OH)D >125 nmol/L was 9.4% (*n* = 318) among the participants who ingested vitamin D supplements, and 0.1% (*n* = 2) among those who ingested vitamin D-fortified foods. The majority (95%) of the prevalence of endpoint serum 25(OH)D >125 nmol/L among the vitamin D supplement RCTs was in those that used doses ≥71 µg/day.

### 3.4. Prevalence of High Serum Calcium Concentrations in Relation to Vitamin D-Fortified Foods and Dietary Supplements in Randomized Controlled Trials

The prevalence of high serum calcium concentrations (>2.50–2.75 mmol/L, based on laboratory-specific values), as an indicator of hypercalcemia, within the RCTs of either vitamin D supplementation or food fortification, and where data were available, are shown in Table 3.

#### 3.4.1. Vitamin D supplementation RCTs

The two subjects from the UK [35] and Finnish [37] trials with elevated serum 25(OH)D concentrations (and total vitamin D intakes were 23.2 and 59.5 µg/day, respectively), mentioned above, had normal calcium concentrations. However, a high serum calcium concentration (>2.65 mmol/L) was evident in two subjects receiving the 20-µg vitamin D_3_ supplement at the endpoint of the RCT in Finland (with total vitamin D intakes 31.5 and 38.0 µg/day, respectively). In three other RCTs within the supplemental dose ≤70 µg/day grouping of trials, high calcium concentrations (>2.55 and 2.60 mmol/L) were found in 0.5–1.6% of subjects, but no cases of serum 25(OH)D >125 nmol/L were evident in the subjects.

In two of the ‘moderately high to high’ dose (≥71 µg/day) RCTs, high calcium concentrations (>2.55 mmol/L) were evident at the endpoint of the trials. In one of the RCTs, one subject receiving 143 µg/day of supplemental vitamin D had high serum calcium (total prevalence 0.4%), while a higher prevalence of 2.9% was observed in the study with a daily dose of 163 µg/day vitamin D + 1000 mg calcium (Table 3). Notably, the prevalence of high serum calcium concentrations among those with serum 25(OH)D >125 nmol/L in that study was 5.6%, while there was no link between high serum calcium concentrations and serum 25(OH)D >125 nmol/L in the other studies.

#### 3.4.2. Vitamin D-Fortified Food-Based RCTs

In the vitamin D_2_-fortified bread study, one subject (2.7%, randomized to 25 µg/day of supplemental vitamin D_2_ in capsule form) had high serum calcium concentrations (>2.65 mmol/L) at the end of the trial [52] (Table 3). Moreover, among the ethnic women who received 20 µg of vitamin D_3_-enriched food (food-based RCT) in Denmark, high serum calcium concentrations (>2.55 mmol/L) were evident in 10.2% of the subjects at the endpoint of the intervention, although, of note, 7.1% had high serum calcium concentrations at baseline (Table 3). The elevated serum calcium concentrations did not change at the endpoint among 2.4% (three subjects) of those with high baseline concentrations, of which 1.6% (two subjects) belong to the placebo group.

Overall, prevalence of high serum calcium was observed in a total of 30 (<1%) out of 3353 participants who either took part in the 20 vitamin D RCTs included in this *ODIN* safety work; in 0.5% (*n* = 16) in the vitamin D supplementation trials; and in 0.4% (*n* = 14) in the studies based on vitamin D-fortified foods.

### 3.5. Potential Additional Adverse Health Effects of High Vitamin D Intake or Serum 25(OH)D Concentrations

#### 3.5.1. Observational Studies: Serum 25(OH)D and Mortality

*ODIN*’s IPD-level meta-analysis of the association between standardized serum 25(OH)D and all-cause, cardiovascular, and cancer mortalities evaluated these at various bands of the serum 25(OH)D concentration [82]. During a median follow-up time of 10.5 years within the eight large European PCSs in older adults, 6802 persons died (total *n* = 26,916). In the context of the safety of high serum 25(OH)D concentrations, there was no apparent excess of mortality (all-cause or CVD-linked) for the group with serum 25(OH)D concentrations >125 nmol/L; albeit, the number of individuals was small (*n* = 172). In addition, there was no significant linear association between 25(OH)D and cancer mortality.

#### 3.5.2. Randomized Controlled Trials of Vitamin D Supplementation and Vitamin D-Enriched Foods

In the *ODIN* supplemental vitamin D RCTs, no serious adverse effects in relation to vitamin D supplementation were reported. An analysis of the data from the pregnancy and birth cohorts showed no evidence of adverse health effects of high S 25(OH) concentrations, which were rare. Similarly, no cases of adverse effects related to the intake of vitamin D-enriched foods were reported in the *ODIN* food-based RCTs. In the *ODIN*’s IPD-level meta-analysis [30], the subgroup analyses, according to the achieved (i.e., endpoint) serum 25(OH)D concentrations, revealed no observed adverse effects of serum 25(OH)D >125 nmol/L arising from vitamin D supplementation on the surrogate markers for cardiovascular disease or glucometabolic health.

## 4. Discussion

Vitamin D toxicity, including hypercalcemia and its sequelae, is rare. However, the adverse health effects, especially those due to the excessive long-term intake of vitamin D, can be serious [4,20]. The IOM has classified circulating 25(OH)D concentrations >125 nmol/L, if sustained, as potentially harmful on the basis of the reported associations with increases in all-cause mortality, a greater risk of cancer at some sites, such as the pancreas, and a greater risk of cardiovascular events, although this level is far lower than the serum 25(OH)D concentrations associated with hypercalcemia, approximately greater than 375–500 nmol/L [15]. Of note, Pilz et al. [4] have emphasized that the risk of adverse events at 25(OH)D concentrations >125 nmol/L has only been inconsistently reported in observational studies, and there are mixed findings in relation to safety from RCTs with subjects having serum 25(OH)D concentrations >125 nmol/L. Recently, safety concerns were raised regarding randomization to supplemental vitamin D at 2000 IU/day (i.e., 50 µg/day), and 4000 IU/day (i.e., 100 µg/day), in the dose-finding phase of a two-stage randomized clinical trial among older adults because of the higher primary outcome rates in terms of falls or death, compared to those assigned 1000 IU/day [82]. This paper provides comprehensive data on the prevalence of standardized serum 25(OH)D >125 nmol/L in a large number of observational studies of children, adults, and older adults in Europe. The prevalence of standardized serum 25(OH)D >125 nmol/L during extended wintertime in the various national surveys and cohorts within *ODIN* was low overall (typically 0.03 to 0.5% in the eight samples that had cases), and, in fact, was absent altogether in the remaining 11 studies. Overall, the prevalence in the combined sample (*n* = 61,082) was 0.1% (*n* = 66). Not unsurprisingly, the prevalence of serum 25(OH)D concentrations >220 nmol/L, which may lead to hypercalcemia [18], was extremely low (<0.005%) in these studies.

The analyses in *ODIN* did not seek to explore the underpinning reasons for the high serum 25(OH)D concentrations in the subjects, but using extended winter as the priority sampling period increases the likelihood that it was induced through the oral ingestion of vitamin D (vitamin D-fortified foods and/or supplements) rather than via UVB-rich sun exposure. A low prevalence of serum 25(OH)D >125 nmol/L has also been reported in other national surveys beyond those included in the present *ODIN* work. For example, data on VDSP standardized serum 25(OH)D >125 nmol/L, from the National Adult Nutrition Survey in Ireland (*n* = 1118), showed a low prevalence of 0.3% (*n* = 3; all sampled in extended summer, and one of the three took vitamin D supplements) [27]. Likewise, in the Finnish nationally representative Health 2011 survey of adults *(n* = 4051), the prevalence of VDSP standardized serum 25(OH)D >125 nmol/L was only 0.2% (i.e., seven participants who used a supplement and one who did not; samples taken within August and December) [83]. An evaluation of data from national nutritional surveys in Europe shows that adults in Finland have the highest mean daily intake of vitamin D from food, including fortified foods, and excluding the contribution from supplements [84].

The prevalence data in the present work is novel as it based on VDSP standardized serum 25(OH)D data, which provides for a truer picture of the prevalence of high serum 25(OH)D in Europe. The standardization of serum 25(OH)D data removes the potential for artefactual high serum concentrations, which are intrinsic to some immunoassays because of the cross-reactivity of the antibodies with other vitamin D metabolites, such as 24,25(OH)2D [28]. Interestingly, the previously reported inverse J-shaped association between high serum 25(OH)D concentrations and all-cause mortality in the NHANES III survey (1988–1994) in the United States, one of the examples referenced by the IOM in issuing their caution about serum 25(OH)D >125 nmol/L, disappeared when the serum 25(OH)D data was standardized [85]. There were only seven individuals with serum 25(OH)D >120 nmol/L when the data was standardized. The *ODIN* IPD-level meta-analysis of eight European cohorts of older adults showed that there was no apparent excess of all-cause or cardiovascular mortality for the group with standardized serum 25(OH)D concentrations >125 nmol/L, acknowledging the limited statistical power due to the small number of individuals with these concentrations (*n* = 172). There is also uncertainty as to whether the reverse J-shaped association between serum 25(OH)D and all-cause mortality is causal. It has been hypothesized that these findings may have been driven by individuals with particularly high 25(OH)D concentrations who started supplementing vitamin D because they were previously vitamin D deficient [85,86,87]. However, *ODIN*’s IPD-level meta-analysis of 12 RCTs, using supplemental vitamin D and stratified according to subgroups of re-measured serum 25(OH)D, showed no adverse effect of the achieved endpoint concentrations >125 nmol/L on the surrogate markers for cardiovascular disease or cardiometabolic health [30].

The data from *ODIN*’s overall collection of 20 vitamin D RCTs, ranging from de novo conducted studies to those in which the serum 25(OH)D was reanalyzed by the project’s CDC-certified method to reduce assay-related differences in estimates for inclusion in the IPD analyses, provided some insight into the relationship between vitamin D supplementation/food fortification and high serum 25(OH)D concentrations. Overall, the prevalence of high serum 25(OH)D was <10% among subjects who received vitamin D supplementation, and <0.1% among those who received foods fortified with vitamin D. However, the incidences of high serum 25(OH)D concentrations were related to the vitamin D dosage. For example, within the nine RCTs of children, adolescents, and adults in Europe, which used supplemental vitamin D_3_ at doses in the range of 5–70 µg/day (as being representative of “low to moderately high” doses), for ≤6 months and generally during an extended winter period, there were only two cases (out of *n* = 1486) with serum 25(OH)D >125 nmol/L. This was also the case for the four winter-based RCTs, which used vitamin D-fortified foods (dose range of 5.5–100 µg/day), with only two cases out of a combined sample of 269. The RCT of vitamin D supplementation during pregnancy had eleven women (out of 144; 7.6%) with serum 25(OH)D >125 nmol/L. These received 10 or 20 µg of vitamin D supplementation for around six months, and while eight of the women had endpoint sampling for serum 25(OH)D in summer, three had endpoint sampling in winter, where UVB-induced synthesis in the skin would not have contributed to the high status. The total vitamin D intakes for these three participants were in the range of 29.6–53.4 µg/day.

Of note, the prevalence of high serum calcium was also very low in these RCTs, with the exception of one of the food-based RCTs, with a prevalence of high serum Ca of 10% at endpoint [54]. In this RCT, however, there may have been methodological issues, as even at baseline there was an unusually high prevalence at 7%. Furthermore, only two participants (1.6%) had serum 25(OH)D >125 nmol/L at endpoint. Overall, these findings are not unexpected, as the total vitamin D intakes in those trials that captured such data were less than the vitamin D UL for children (Denmark) [34], adolescents (UK) [35], pregnant women (Ireland) [36], women with Danish and Pakistani origin in Denmark [54], and women with Finnish and East African backgrounds in Finland [37,52].

On the other hand, a high prevalence of serum 25(OH)D >125 nmol/L was evident in each of the six RCTs that administered “moderately high to high” doses of vitamin D_3_ (≥71 µg/day). Overall, 21.0% (*n* = 307 out of 1459) had endpoint serum 25(OH)D >125 nmol/L. It was particularly evident in the trials that used doses of supplemental vitamin D >140 µg/day, where between 31.8 and 91.9% of the participants had serum 25(OH)D >125 nmol/L at endpoint. All six RCTs were of 6 to 12 months in duration and, thus, would have had some summer blood sampling. However, in general, the prevalence of high serum 25(OH)D in the placebo groups was absent or very low, suggesting oral intake as the main driver of high status. These data are also of note, all were reanalyzed serum 25(OH)D using the project’s certified LC-MS/MS method, avoiding the inflated estimates that can occur with some methods [28]. More recently, Burt and coauthors, in their three-year vitamin D RCT, also observed the occurrence of serum 25(OH)D >125 nmol/L in those arms with supplemental vitamin D doses of 100 and 250 µg/d, but not in the arm with a dose of 10 µg/day [88]. A recent systematic review and meta-analysis demonstrated consistent dose–response relationships between serum 25(OH)D concentrations (from a variety of different analytical methods) and vitamin D supplementation (dose range of 10–149 µg/day), among children, adolescents, and adults (including pregnant and lactating women, postmenopausal women, and the elderly) from around the globe. It also highlights cases of high serum 25(OH)D concentration among the highest supplemental doses (>100 µg/day) [89].

Three out of the five “moderately high to high-dose” vitamin D RCTs that had data reported no prevalence of high serum calcium concentrations. In the remaining two RCTs [47,48], high serum calcium concentrations (>2.55 mmol/L) were observed at the endpoint of the trials (six months and one year, respectively). The highest prevalence of elevated serum calcium (3%; *n* = 8 out of 275) was seen in the study in which a daily dose of 1000 mg of calcium was administered, together with a daily vitamin D_3_ dose of 163 µg/day [48]. Notably, the prevalence of high serum calcium among those with serum 25(OH)D >125 nmol/L in that study was 6%, while there was no link between elevated serum calcium and serum 25(OH)D >125 nmol/L in the other studies. In the RCT of Burt et al., where the effect of high-dose vitamin D supplementation on bone mineral density was investigated for three years, episodes of hypercalcemia and hypercalciuria (in the range of 4–9% and 22–33%, respectively) were found with the doses of 100 and 250 µg/day, respectively [88]. While there were cases of hypercalciuria (17%, but not hypercalcemia) with the vitamin D dose of 10 µg/d in the study, these were possibly linked to the calcium intake, which was based on achieving 1200 mg/day [88]. A systematic review and meta-analysis of long-term studies (*n* = 37) of vitamin D supplementation in adults showed an increased risk of hypercalcemia in vitamin D supplementation groups [90]. Subgroup analyses showed that the risk was not modified by the vitamin D dose (≤20 [*n* = 3 studies] or >20 µg/day [*n* = 33 studies]) and meta-regression showed no association between the vitamin D dose and the risk of hypercalcemia [90]. In contrast, the present findings seem to point to a high dose of vitamin D supplements in relation to a trend for elevated serum calcium. From a wider safety perspective, it should also be noted again that high endpoint serum 25(OH)D were not related to increases in surrogate markers for cardiovascular disease or cardiometabolic health in the *ODIN* IPD of the RCTs using supplemental vitamin D [30]. While this analysis included data from 12 RCTs, only the 6 high-dose trials had participants with serum 25(OH)D >125 nmol/L [30].

In efforts to prevent vitamin D deficiency and improve vitamin D status in the population, national vitamin D fortification policies have been established in some countries, especially at the high latitudes (e.g., United States, Canada, Finland, Norway, and Sweden) [91]. In those countries, usually fluid milk products and/or fat spreads have been fortified [91]. In other European member states, the voluntary fortification of some food products is practiced, but the impact of this mode of fortification on the population intakes of vitamin D has been shown to be modest at best [92].

In further support of the good safety profile of vitamin D-fortified foods, the findings of low prevalence of high endpoint serum 25(OH)D concentrations, and elevated serum calcium in the *ODIN* RCTs, with no evidence of adverse health effects, showed that these vitamin D-fortified food products (in addition to fortified dairy products) can effectively, safely, and sustainably increase habitual vitamin D intakes, and prevent deficiency in the general population.

The main strengths of this work are that our data are based on a large collection of observational studies representing a wide range of European populations (infants, children, adolescents, adults, older adults, pregnant women, and immigrants). Importantly, the serum 25(OH)D data from these studies was standardized, enhancing the comparability across the studies [26] and limiting the impact of method-related artefactually elevated serum 25(OH)D concentrations [28]. This is also the first study, to the best of our knowledge, to report high serum 25(OH)D concentrations in a selection of RCTs, offering the highest level of scientific evidence for causality. In the case of the 20 vitamin D RCTs, serum 25(OH)D was analyzed, or, specifically, reanalyzed, by the project’s certified method. This has benefits not only for the true prevalence of serum 25(OH)D >125 nmol/L, but also for decreasing the well-established method-related variability arising from, and inherent in, IPD analyses. Nevertheless, our collection of studies within European countries limits the generalizability of our findings to other populations. Other limitations of this study include incomplete data on urinary calcium concentrations and hypercalciuria. Serum calcium concentrations were not available in some RCTs, and, for others, it was not stratified by intervention groups. Moreover, the absence of data on dietary vitamin D intakes from the majority of the cohort studies was a limitation in examining the relationship between the total vitamin D intakes and the prevalence of high endpoint serum 25(OH)D concentrations.

## 5. Conclusions

A consideration of all safety aspects of food-based solutions for addressing vitamin D deficiency is paramount prior to initiating public health measures. From a safety perspective, the data from across the *ODIN* project suggest that high serum 25(OH)D concentrations (i.e., >125 nmol/L) are relatively rare in European populations. However, the risk of high serum 25(OH)D can be increased with high-dose vitamin D supplementation (in excess of 70 µg/day (2800 IU)). In addition, high-dose vitamin D supplement use also increases the risk of exceeding the EFSA-defined UL for vitamin D, the touchstone public health safety index. The inclusion of vitamin D-(bio)fortified foods, singularly, but also in combination (which has the most benefit in terms of the prevention of vitamin D inadequacy) carries little or no risk of exceeding the UL for vitamin D, even when low-dose vitamin D supplements, up to the recommended intake value, are used in tandem.

## Figures and Tables

**Table 1:** Characteristics of the included randomized controlled trials within *ODIN* safety work.

References	Country	Total *n*	Age (Yrs)	Sex: % Female	Population Group	Duration of Intervention	Intervention Groups
Cashman et al., 2009 *, Muldowney et al., 2012 *	Ireland	200	≥64	59.2	Adults	22 wk	Placebo-controlled
							Vitamin D_3_ Supplements (5 or 10 or 15 µg/d)
Cashman et al., 2008 *, Muldowney et al., 2012 *	Ireland	214	20–40	50.0	Adults	22 wk	Placebo-controlled
							Vitamin D_3_ Supplements (5 or 10 or 15 µg/d)
Mortensen et al., 2016 **	Denmark	119	4–8	53.1	Children	20 wk	Placebo-controlled
							Vitamin D_3_ Supplements (10 or 20 µg/d)
Smith et al., 2016 **	UK	105	14–18	57.3	Adolescents	20 wk	Placebo-controlled
							Vitamin D_3_ Supplements (10 or 20 µg/d)
O’Callaghan et al., 2018 **	Ireland	144	21–41	100	Pregnant women	25 wk	Placebo-controlled
							Vitamin D_3_ Supplements (10 or 20 µg/d)
Adebayo et al., 2018 **	Finland	125	21–64	100	Ethnic women	5 mo	Placebo-controlled
							Vitamin D_3_ Supplements (10 or 20 µg/d)
Chel et al., 2008 *	Netherlands	273	>70 years	77.4	Nursing home residents	4 mo	Placebo-controlled
							Vitamin D_3_ Supplements (15 µg/d)
Wicherts et al., 2011 *	Netherlands	148	18–65	74.8	Non-western immigrants, 25(OH)D <25 nmol/L	6 mo	Placebo-controlled
							Vitamin D_3_ Supplements (20 µg/d)
Oosterwerff et al., 2014 *	Netherlands	110	20–65	60.0	Non-western immigrants, prediabetic, with 25(OH)D <50 nmol/L	16 wk	Placebo-controlled
							Vitamin D_3_ Supplements (30 µg/d)
Pilz et al., 2015 *	Austria	187	≥18	47.0	Persons with history of arterial hypertension, 25(OH)D <75 nmol/L	8 wk	Placebo-controlled
							Vitamin D_3_ Supplements (70 µg/d)
Sollid et al., 2014 *	Norway	484	21–80	38.6	Persons with IGT and/or IFG	1yr	Placebo-controlled
							Vitamin D_3_ Supplements (71 µg/d)
Sneve et al., 2008 *, Jorde et al., 2010 *, Beilfuss et al., 2012 *	Norway	334	21-70	64.2	Persons with high BMI	1 yr	Placebo-controlled
							Vitamin D_3_ Supplements (71 or 143 µg/d)
Grimnes et al., 2011 *	Norway	93	30–75	49.5	Persons with 25(OH)D <42 nmol/L	6 mo	Placebo-controlled
							Vitamin D_3_ Supplements (143 µg/d)
Kjaergaard et al., 2012 *	Norway	230	30–75	54.7	Persons with 25(OH)D <55 nmol/L	6 mo	Placebo-controlled
							Vitamin D_3_ Supplements (143 µg/d)
Grimnes et al., 2012 *	Norway	275	50–80	100	Women with low BMD	1 yr	Placebo-controlled
							Vitamin D_3_ Supplements (163 µg/d)
Wamberg et al., 2013 * Wamberg et al., 2013 *	Denmark	43	18–50	71.2	Persons with high BMI, 25(OH)D <50 nmol/L	6 mo	Placebo-controlled
							Vitamin D_3_ Supplements (175 µg/d)
Urbain et al., 2011 *	Germany	26	≤45	65	Adults	4 wk	Placebo-controlled
							Vitamin D_2_-enriched mushrooms or D_2_ supplement (700 µg/wk)
Itkonen et al., 2016 **	Finland	37	20–37	100	Adults	8 wk	Placebo-controlled
							Vitamin D_2_-enriched bread or D_2_ supplement (25 µg/d) or D_3_ (25 µg/d)
Manios et al., 2017 **	Greece	79	55–75	100	Adults	20 wk	Placebo-controlled
							Vitamin D_3_-enriched Gouda cheese (5.7 µg/d)
Grønborg et al., 2020 **	Denmark	127	18–50	100	Ethnic women	3 mo	Placebo-controlled
							Vitamin D_3_-enriched food (20 µg/d)

* Reanalyzed 25(OH)D; ** 25(OH)D analyzed de novo; µg/d, µg/day; IGT, impaired glucose tolerance; IFG, impaired fasting glucose; BMI, body mass index; BMD, bone mineral density; wk, weeks; mo, months; yr, year.

**Table 2:** Prevalence of serum 25-hydroxyvitamin D (S-25(OH)D) >125 nmol/L at baseline and at endpoint in *ODIN* RCTs, stratified by vitamin D intervention and dose.

References	Population Group	Duration of Intervention	Intervention Groups	Prevalence (%) of S-25(OH)D >125 nmol/L (*n*/Total *n*)	
				Baseline	Endpoint
**Vitamin D supplementation RCTs—Supplemental vitamin D_3_ dose ≤70 µg/d**					
Cashman et al., 2009 *, Muldowney et al., 2012 *	Persons >63 years of age	22 wk	0 µg of vitamin D_3_	0	0
			5 µg of vitamin D_3_	0	0
			10 µg of vitamin D_3_	0	0
			15 µg of vitamin D_3_	0	0
Cashman et al., 2008 *, Muldowney et al., 2012 *	Persons 20–40 years of age	22 wk	0 µg of vitamin D_3_	1.8 (1/56)	0
			5 µg of vitamin D_3_	0	0
			10 µg of vitamin D_3_	1.8 (1/57)	0
			15 µg of vitamin D_3_	1.9 (1/52)	0
Mortensen et al., 2016 **	Children	20 wk	0 µg of vitamin D_3_	0	0
			10 µg of vitamin D_3_	0	0
			20 µg of vitamin D_3_	0	0
Smith et al., 2016 **	Adolescents	20 wk	0 µg of vitamin D_3_	0	0
			10 µg of vitamin D_3_	0	0
			20 µg of vitamin D_3_	0	2.6 (1/38)
O’Callaghan et al., 2018 **	Pregnant women	25 wk	0 µg of vitamin D_3_	0	0
			10 µg of vitamin D_3_	0	13.5 (5/37)
			20 µg of vitamin D_3_	0	13.6 (6/44)
Adebayo et al., 2018 **	Women of East African descent	5 mo	0 µg of vitamin D_3_	0	0
			10 µg of vitamin D_3_	0	0
			20 µg of vitamin D_3_	0	8.3 (1/12)
	Women of Finnish descent		0 µg of vitamin D_3_	0	0
			10 µg of vitamin D_3_	0	0
			20 µg of vitamin D_3_	0	0
Chel et al., 2008 *	Nursing home residents >70 years of age	4 mo	0 µg of vitamin D_3_	0	0
			15 µg of vitamin D_3_	0	0
Wicherts et al., 2011 *	Non-western immigrants with 25(OH)D values <25 nmol/L	6 mo	0 µg of vitamin D_3_	0	0
			20 µg of vitamin D_3_	0	0
Oosterwerff et al., 2014 *	Non-western immigrants with pre-diabetes and 25(OH)D values <50 nmol/L	16 wk	0 µg of vitamin D_3_	0	0
			30 µg of vitamin D_3_	0	0
Pilz et al., 2015 *	Persons with a history of arterial hypertension and 25(OH)D values <75 nmol/L	8 wk	0 µg of vitamin D_3_	0	0
			70 µg of vitamin D_3_	0	0
**Vitamin D supplementation RCTs—Supplemental vitamin D_3_ dose ≥71 µg/d**					
Sollid et al., 2014 *	Persons with IGT and/or IFG	1 yr	0 µg of vitamin D_3_	0.4 (1/255)	0.8 (2/242)
			71 µg of vitamin D_3_	0.4 (1/256)	7.0 (17/242)
Sneve et al., 2008 *, Jorde et al., 2010 *, Beilfuss et al., 2012 *	Persons with a high BMI	1 yr	0 µg of vitamin D_3_	0	0
			71 µg of vitamin D_3_	0	11.3 (12/106)
			143 µg of vitamin D_3_	0	53.4 (62/116)
Grimnes et al., 2011 *	Persons with 25(OH)D values <42 nmol/L	6 mo	0 µg of vitamin D_3_	0	0
			143 µg of vitamin D_3_	0	46.9 (23/49)
Kjaergaard et al., 2012 *	Persons with 25(OH)D values <55 nmol/L	6 mo	0 µg of vitamin D_3_	0	0
			143 µg of vitamin D_3_	0	49.2 (59/120)
Grimnes et al., 2012 *	Women with a low BMD	1 yr	0 µg of vitamin D_3_	0.7 (1/148)	0
			163 µg of vitamin D_3_	1.3 (2/149)	91.9 (125/136)
Wamberg et al., 2013 * Wamberg et al., 2013 *	Persons with a high BMI and 25(OH)D values <50 nmol/L	6 mo	0 µg of vitamin D_3_	3.8 (1/26)	0
			175 µg of vitamin D_3_	0	31.8 (7/22)
**Vitamin D fortified food based RCTs**					
Urbain et al., 2011 *	Adults	4 wk	Placebo-controlled	0	0
			D_2_-enriched mushrooms providing 700 µg of vitamin D_2_ weekly	0	0
			D_2_ supplement providing 700 µg of vitamin D_2_ weekly	0	0
Itkonen et al., 2016 **	Adults	8 wk	Placebo-controlled	0	0
			D_2_-enriched bread providing 25 µg of vitamin D_2_ daily	0	0
			D_2_ supplement providing 25 µg of vitamin D_2_ daily	0	0
			D_3_ supplement providing 25 µg of vitamin D_3_ daily	0	0
Manios et al., 2017 **	Adults	20 wk	Placebo-controlled	0	0
			D_3_-enriched Gouda cheese providing 5.7 µg of vitamin D_3_ daily	0	0
Grønborg et al., 2020 **	Women of Pakistani descent	3 mo	Placebo-controlled	0	0
			D_3_-enriched food providing 20 µg of vitamin D_3_ daily	2.9 (1/35)	2.9 (1/35)
	Women of Danish descent		Placebo-controlled	0	0
			D_3_-enriched food providing 20 µg of vitamin D_3_ daily	0	2.7 (1/37)

S-25(OH)D, serum 25-hydroxyvitamin D concentration; RCTs, randomized controlled trials; * Reanalyzed 25(OH)D; ** 25(OH)D analyzed de novo; µg/d, µg/day; IGT, impaired glucose tolerance; IFG, impaired fasting glucose; BMI, body mass index; BMD, bone mineral density; wk, weeks; mo, months; yr, year.

**Table 3:** Prevalence of high serum calcium (S-Ca) concentrations in *ODIN* RCTs.

References	Population Group	Type of Intervention	Upper Limit (UL) of Reference Range for S-Ca (mmol/L)	Number of Subjects with S-Ca >UL Out of Total Number of Subjects	Prevalence (%) of Subjects Exceeding Upper Limit of Reference Range of S-Ca	Highest S-Ca Concentration mmol/L if >UL
**Vitamin D supplementation RCTs—Supplemental vitamin D_3_ dose ≤70 µg/d**						
Cashman et al., 2009 *, Muldowney et al., 2012 *	Persons of age > 63 yrs	Vitamin D_3_	2.60	1/200	0.5	NA
Cashman et al., 2008 *, Muldowney et al., 2012 *	Persons of age 20–40 yrs	Vitamin D_3_	2.60	0/214	0	NA
Mortensen et al., 2016 **	Children	Vitamin D_3_	>2.70	0	0	
Smith et al., 2016 **	Adolescents	Vitamin D_3_	2.50	0	0	
O’Callaghan et al., 2018 **	Pregnant women	Vitamin D_3_	2.63	0	0	0
Adebayo et al., 2018 **	Ethnic women	Vitamin D_3_	2.65	9/147 (baseline) 2/125 (endpoint)	6.1 (baseline) 1.6 (endpoint)	2.82 (baseline) 2.74 (endpoint)
Chel et al., 2008 *	Nursing home residents, age >70 years	Vitamin D_3_	NA	NA	NA	NA
Wicherts et al., 2011 *	Non-western immigrants, 25(OH)D <25 nmol/L	Vitamin D_3_	2.60	1/112	0.9	NA
Oosterwerff et al., 2014 *	Non-western immigrants, prediabetic, with 25(OH)D <50 nmol/L	Vitamin D_3_ ***	2.60	0/110	0	NA
Pilz et al., 2015 *	Persons with history of arterial hypertension, 25(OH)D <75 nmol/L	Vitamin D_3_	2.55	3/188	1.6	NA
**Vitamin D supplementation RCTs—Supplemental vitamin D_3_ dose ≥71 µg/d**						
Sollid et al., 2014 *	Persons with IGT and/or IFG	Vitamin D_3_ ***	2.60	0/484	0	NA
Sneve et al., 2008 *, Jorde et al., 2010 *, Beilfuss et al., 2012 *	Persons with high BMI	Vitamin D_3_	2.60	0/334	0	NA
Grimnes et al., 2011 *	Persons with 25(OH)D <42 nmol/L	Vitamin D_3_	2.60	0/94	0	NA
Kjaergaard et al., 2012 *	Persons with 25(OH)D <55 nmol/L	Vitamin D_3_	2.55	1/230	0.4	NA
Grimnes et al., 2012 *	Women with low BMD	Vitamin D_3_ ***	2.55	8/275	2.9	NA
Wamberg et al., 2013 * Wamberg et al., 2013 *	Persons with high BMI, 25(OH)D <50 nmol/L	Vitamin D_3_	NA	NA	NA	NA
**Vitamin D fortified food based RCTs**						
Urbain et al., 2011 *	Adults	D_2_-enriched mushrooms	>2.70	0	0	
Itkonen et al., 2016 **	Adults	D_2_-enriched bread, vitamin D_2_ and D_3_ supplements	2.65	1/37 (endpoint)	2.7 (endpoint)	2.86 (endpoint)
Manios et al., 2017 **	Adults	D_3_-enriched Gouda cheese	NA	NA	NA	NA
Grønborg et al., 2020 **	Ethnic women	D_3_-enriched food	2.55	9/127 (Baseline) 13/127 (endpoint)	7.1 (baseline) 10.2 (endpoint)	2.67 (Baseline)

S-Ca, serum calcium concentration; RCTs, randomized controlled trials; * Reanalyzed RCT with endpoint data only; ** 25(OH)D analyzed de novo; µg/d, µg/day; IGT, impaired glucose tolerance; IFG, impaired fasting glucose; BMI, body mass index; BMD, bone mineral density; *** Intervention groups receiving calcium; NA, not available.

## Data Availability

Data is contained within the article and/or its supplementary material. Specific request in relation to data beyond that presented should be made to the PI (CL-A) for consideration.

## Supplementary Materials

The following are available online at https://www.mdpi.com/article/10.3390/foods10123065/s1, Figure S1: The work packages (WP) within the *ODIN* vitamin D project and their workflows and interdependencies. WP1-8 reported all their safety data to WP9 as the dedicated safety WP; Table S1: Tolerable upper intake levels (ULs) for vitamin D; Table S2: Included national nutrition surveys and epidemiological cohorts/samples of European populations and prevalence of high serum 25-hydroxyvitamin D (25(OH)D) concentrations (>125 nmol/L).
