# Supplementary material for: Safety of Vitamin D Food Fortification and Supplementation: Evidence from Randomized Controlled Trials and Observational Studies
Source: Foods. 2021 Dec 9;10(12):3065. doi: 10.3390/foods10123065 (PMC8701201; doi:10.3390/foods10123065)
Supplement: Supplementary file 1 [file foods-10-03065-s001.zip › foods-1458487-supplementary.pdf]

foods

MDPI

Article

Safety of Vitamin D Food Fortification and Supplementation: Evidence from Randomized Controlled Trials and Observational Studies

Folasade A. Adebayo [1], Suvi T. Itkonen [1], Taina Öhman [1], Mairead Kiely [2], Kevin D. Cashman [2], Christel Lamberg-Allardt [1,*] and on behalf of the ODIN consortium †

[1] Calcium Research Unit, Department of Food and Nutrition, University of Helsinki, Helsinki, P.O. Box 66, FI-00014, Finland; folasade.adebayo@helsinki.fi (F.A.A.); suvi.itkonen@helsinki.fi (S.T.I); taina.ohman@gmail.com (T.Ö)
[2] Cork Centre for Vitamin D and Nutrition Research, School of Food and Nutritional Sciences, University College Cork, T12 Y337 Cork, Ireland; M.Kiely@ucc.ie (M.K.); K.Cashman@ucc.ie (K.D.C.)
* Correspondence: christel.lamberg-allardt@helsinki.fi
† the ODIN Consortium are listed in acknowledgments.

Supplementary data:

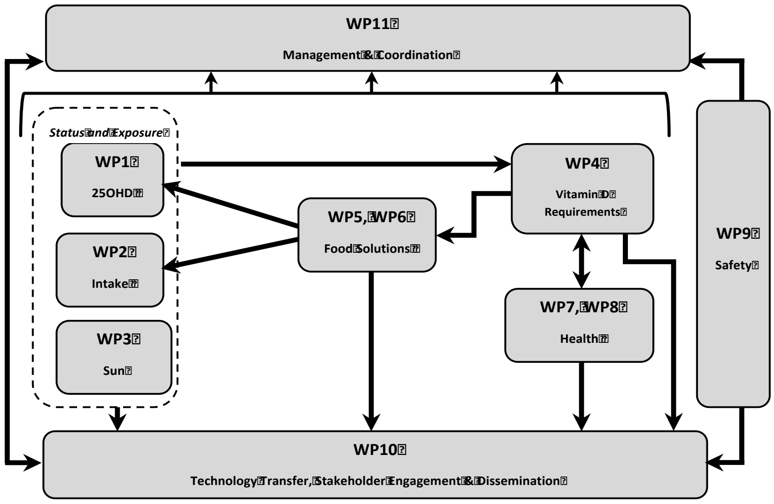

Figure S1. The work packages (WP) within the ODIN vitamin D project and their work-flow and interdependencies. WP1-8 reported all their safety data to WP9 as the dedicated safety WP.

Table S1. Tolerable upper intake levels (ULs) for vitamin D.

	**Vitamin D UL recommendations**	
**Age***	***IOM (USA)***	***EFSA (EU)***
0-6 months	25 µg (1,000 IU)	25 µg (1,000 IU)**
6/[7]–12 months	38 µg (1,500 IU)	35 µg (1,500 IU)**
1–3 years	63 µg (2,500 IU)	50 µg (2,500 IU)
4–8/[10] years	75 µg (3,000 IU)	50 µg (3,000 IU)
9/[11]–[17]/18 years	100 µg (4,000 IU)	100 µg (4,000 IU)
[18]/19+ years	100 µg (4,000 IU)	100 µg (4,000 IU)
Pregnancy	100 µg (4,000 IU)	100 µg (4,000 IU)
Lactation	100 µg (4,000 IU)	100 µg (4,000 IU)

IOM, Institute of Medicine; EFSA, European Food Safety Authority. *Ages shown in brackets reflect where EFSA had differences in age bracket to that of IOM. **EFSA, 2018, all other from EFSA 2012

foods MDPI

Table S2. Included national nutrition surveys and epidemiological cohorts/samples of European populations and prevalence of high serum 25-hydroxyvitamin D (25(OH)D) concentrations (>125 nmol/L).

Type of Study	Country	Included surveys	*n*	% of S-25(OH)D >125 nmol/L*		References
				Yearly	*Extended winter***	
**Pooled analysis of 20 surveys with various populations**	9 European countries	Healthy Lifestyle in Europe by Nutrition in Adolescence (HELENA) study	1006	0	0	Moreno et al. 2008, González-Gross et al. 2012
	Denmark	The Optimal well-being, development and health for Danish children through a healthy New Nordic Diet (OPUS) School Meal Study	779	0	0	Damsgaard et al. 2012, Damsgaard et al. 2014
	Norway	The Tromsø Study: Fit Futures	890	0	0	Oberg et al. 2014, Winther et al. 2014, The Tromsø Study 2021
	Greece	The Healthy Growth Study (HGS)	806	0	0	Moschonis et al. 2010
	Greece	The Infant's Nourishment and Nutritional Status (INNS) study	222	0	0	McBride et al. 2012
	Ireland	The Cork BASELINE birth cohort study	742	0.4	0.5	Kiely et al. 2017, Ni Chaoimh et al. 2018
	Germany	The German Health Interview and Examination Survey for Children and Adolescents (KIGGS)	10,015	0	0	Kurth et al. 2008
	United Kingdom	National Diet and Nutrition Survey (NDNS): Years 1-4 (combined) of the Rolling Programme (2008/2009 – 2011/12)	511+977	0	0	National Diet and Nutrition Survey 2014
	Germany	The German Health Interview and Examination Survey for Adults (DEGS) ***	6995	0	0	Scheidt-Nave et al. 2012, Kamtsiuris et al. 2013
	Norway	The Tromsø study - 6th Survey (Tromsø 6)	12,817	0.6	0.3	Grimnes et al. 2010, Jacobsen et al. 2012, Eggen et al. 2013, The Tromsø Study 2021
	Netherlands	The New Hoorn Study (NHS) ***	2625	0.4	0.3	van 't Riet et al. 2010
	Netherlands	Longitudinal Aging Study Amsterdam (LASA) 2009	915	0.7	0	Huisman et al. 2011
	Iceland	The Age, Gene/Environment Susceptibility (AGES-Reykjavik) study***	5519	0	0	Harris et al. 2007
	Finland	The Finnish Migrant Health and Wellbeing Study (Maamu)	1310	0.3	0.2	Tiittala et al. 2015, Finnish Institute for Health and Welfare 2021

Table S2 continued…

Type of Study	Country	Included surveys	*n*	% of S-25(OH)D >125 nmol/L*		References
				Yearly	*Extended winter***	
**Pooled analysis of two pregnant cohorts**	Ireland	The SCOPE Ireland pregnancy cohort study	1786	0.5	0	Kiely et al. 2016
	Sweden	The GraviD cohort	2079	0.4	0	Bärebring et al.2016
**Meta-analysis of eight older adults cohorts**	Germany	Ludwigshafen RIsk and Cardiovascular Health (LURIC) Study	3299	0.4	0.2	Gaksch et al. 2016
	Denmark	Aarhus Mammography Cohort Study	2473	0.6	0.3	
	Norway	The Tromsø study - 4th Survey (Tromsø 4)	7145	0.03	0.03	
	Netherlands	Longitudinal Aging Study Amsterdam (LASA) 1995	1302	0	0	
	Netherlands	Longitudinal Aging Study Amsterdam (LASA) 2002	734	0.1	0.1	

*Percentages rounded; **October to March; ***Also included in meta-analysis of eight older adults cohort
